# Survival Following Extreme Hypernatraemia Associated with Severe Dehydration and Undiagnosed Diabetes Mellitus

**DOI:** 10.1155/2019/4174259

**Published:** 2019-12-12

**Authors:** Hao Xiao, Rahul Barmanray, Sarah Qian, Dilantha De Alwis, Gerard Fennessy

**Affiliations:** ^1^Department of Intensive Care, Western Health, Melbourne, VIC, Australia; ^2^Department of Diabetes and Endocrinology, Western Health, Melbourne, VIC, Australia; ^3^Department of Diabetes and Endocrinology, The Royal Melbourne Hospital, Melbourne, VIC, Australia

## Abstract

We report a case of a previously well 58-year-old man, who presented with delirium and low GCS, and was found to have extreme hypernatraemia (Na^+^ = 191 mmol/L) and hyperglycaemia (glucose = 31 mmol/L). This resulted in a corrected serum sodium of 202 mmol/L. He was treated with fluid and electrolyte replacement in the intensive care unit, and had returned to essentially normal function by hospital discharge. The aetiology was believed to be due to severe dehydration and a new diagnosis of diabetes mellitus. Extreme hypernatraemia (serum sodium level greater than 190 mmol/L) is rare and associated with a high mortality. The mainstay of treatment is careful fluid and electrolyte management. Most recommendations advise to reduce the serum sodium by 0.5 mmol/L/hour, due to concerns over cerebral oedema; however, there are reports that slower correction is associated with higher mortality. In this case, the initial corrected sodium of 202 mmol/L was steadily corrected to 160 mmol/L over 91 hours, at a rate of 0.46 mmol/L/hour. This demonstrates the safety of the recommended approach.

## 1. Introduction

Hypernatraemia is defined as a serum sodium (Se[Na^+^]) greater than 145 mmol/L, with severe and extreme hypernatraemia defined as Se[Na^+^] being greater than 160 and 190 mmol/L, respectively. Hypernatraemia can occur either due to excess sodium-containing solute gain (e.g., deliberate ingestions) or, more often, nonreplaced water losses, particularly from the gastrointestinal or renal tracts. Severe hypernatraemia is associated with neurological deficits and carries a mortality of 37–60% [1]. Mortality increases with a higher initial serum sodium [2]. There are few reported adult cases with extreme hypernatraemia (Se[Na^+^] > 190 mmol/L) at admission. We present a case of extreme hypernatraemia, associated with marked dehydration and hyperglycaemia in an adult with a corrected Se[Na^+^] of 202 mmol/L at presentation. Following a period of observation and careful fluid and electrolyte management in the Intensive Care Unit (ICU), the serum sodium returned to normal. The patient was discharged back to the community after a period in rehabilitation.

## 2. Case Presentation

A 58-year-old man presented to Sunshine hospital, a major tertiary hospital in the west of Melbourne, Victoria, Australia, with altered mental state, Se[Na^+^] of 191 mmol/L and Se[Glc]) 31 mmol/L (558.6 mg/dL). He had been found outside where the ambient temperature had been recorded as 34.2°C (93.5°F). Although he had no significant past medical history, he had presented to the emergency department of the same hospital seven days prior with complaints of mild abdominal pain. No formal diagnosis was made, and he had been discharged to his general practitioner for follow-up. Investigations at this stage showed Se[Na^+^] 141 mmol/L, and Se[Glc] 6 mmol/L. Collateral history confirmed that the patient had been well following his previous discharge, attended work every day, and appeared to have been normal thirteen hours prior to presentation, mowing the lawn. He had been found collapsed inside and unable to get up from the floor. He was partially clothed and making incoherent sounds.

On initial assessment in the emergency department, his Glasgow Coma Scale was 11 (E4 V1 M6). His blood pressure was 100/60 mmHg, heart rate 120 beats per minute, and temperature 36.2°C. His weight was 54.1 kg, and his mucous membranes were dry.

Initial laboratory tests showed a Se[Na^+^] 191 mmol/L and Se[Glc] of 31 mmol/L. The corrected sodium was calculated using Hillier's equation (Corrected sodium = measured sodium + 0.024 × (serum glucose −100)), giving a value of Se[Na^+^] 202 mmol/L [3]. Initial serum osmolality was 445 mOsmol/kg, paired urine osmolality was 811 mOsm/kg, and urine sodium was 41 mmol/L. Hba1c was 9.9% (85 mmol/mol). Other biochemistry results are reported in Table 1. The patient was admitted to the ICU and had an intra-arterial line and urinary catheter inserted.

Although the onset of hypernatraemia was not clear, a normal sodium 7 days prior and reported normal function 13 hours earlier supported that the serum sodium rise was likely to be relatively acute, and a sodium correction rate of 0.5 mmol/L/hour was adopted. Arterial blood gas samples (ABGs) were taken hourly for the first 48 hours, specifically tracking sodium, chloride, and glucose. Because the sodium/chloride gap was extreme (48 mmol/L), fluid resuscitation for the first 48 hours consisted of both 0.9% saline (5500 ml) and compound sodium lactate (11,500 ml). 5% dextrose and insulin infusion was subsequently added after first 24 hours.

**Figure 1:**
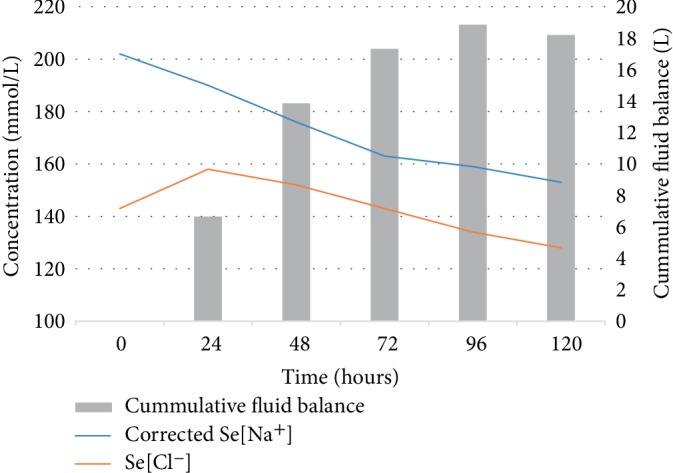
Serum sodium and fluid balance during treatment.

After 72 hours, the corrected sodium reached 168, and the serum sodium correction rate was 0.42, 0.50, and 0.67 mmol/L/hour for the three 24-hour periods following admission. Once the serum sodium level was under 160 mmol/L, the frequency of investigations dropped and strict fluid management was relaxed. The corrected serum sodium reached below 145 after about 7 days (Figure 1).

Using Adrogue's equation, the estimated water deficit was 11.8 L (Free water deficit = 60% × weight × (current sodium/ideal sodium−1)) and 2.7 L of fluid was given in the first 12 hours (400 ml normal saline total in first 2 hours and 600 ml normal saline total in first 4 hours) [4]. Urine output was 1.95 L in the first 48 hours.The patient's GCS improved to 15 on day 4. He was discharged from ICU to the endocrinology ward. His weight had increased from 54.1 kg on admission to 72.5 kg on day 9.—A gain of 18.4 kg. The cumulative fluid balance chart recorded a positive balance of 22.3 L. Hence approximately 3.9 litres (or 433 ml/day) was accounted for by insensible losses.

Follow-up MRI showed a normal intracranial structure and pituitary gland, while pituitary biochemical function was normal. Following ICU discharge the patient exhibited a fluctuating delirium with a Rowland Universal Dementia Assessment Scale (RUDAS) score of 20 out of 30 prior to transfer to the rehabilitation ward [10]. On discharge to the community two months later, the patient had returned almost to baseline functional status, with the exception of a mild attentional deficit. He was performing all his personal, domestic, and community activities of daily living as he had been prior to admission, except for driving.

## 3. Discussion

Severe hypernatraemia is associated with significant mortality and neurological impairment [1]. There are few reported cases of extreme hypernatraemia, and they are commonly associated with salt poisoning or diabetes insipidus [6, 7]. In the context of no known history of salt ingestion, elevated urine osmolality and HbA1c, and rapid deterioration, our patient's presentation is likely a result of dehydration related to both excessive perspiration in high ambient temperatures and glycosuria from undiagnosed diabetes mellitus. It was felt to be unusual that an otherwise cognitively well middle-aged man was unable to self-treat dehydration with water ingestion; however, investigations looking for neurological or toxic pathology were unrevealing.

Management of extreme hypernatraemia is challenging. In theory, overly rapid correction of hypernatraemia can cause intracellular water movement into neuronal cells, causing cerebral edema. This has been well-documented in paediatric cases and animal studies [4, 5]. Current recommendations for correction of chronic hypernatraemia (>48 hours) include a maximum Se[Na^+^] correction rate of 0.5 mmol/L/hour or 10–12 mmol per 24 hours [4, 6].

Acute hypernatraemia may be safely corrected more rapidly. Indeed, several cases report no evidence of cerebral edema despite rapid sodium correction [7, 8]. Alshayeb et al. report that in a cohort of 117 patients with hypernatraemia, (mean initial sodium of 159 mmol/L), faster correction of sodium carried a lower mortality [2]. In this study 30-day mortality for a correction rate of ≥0.25 mmol/L/hour was 18% compared with 46% for a rate of <0.25 mmol/L/hour. The fastest 24-hour correction rate was 1.38 mmol/L/hour. This supports the safety of more rapid sodium correction in cases of acute hypernatraemia cases. It is notable that the mean sodium correction rate in our patient over the 120 hours to eunatraemia was 0.475 mmol/L/hour, though the fastest rate of reduction over any 24-hour period was 0.625 mmol/L/hour (over a single 24-hour period), thus demonstrating the safety of the recommended 0.5 mmol/L/hour reduction.

If hypernatraemia is coexistent with hyperglycaemia, this must be considered, including correcting Serum sodium measurements. This allows for the sodium-lowering effect of fluid rehydration to be differentiated from the increase in sodium that accompanies correction of hyperglycaemia [3]. Paralleling hypernatraemia correction principles, hyperglycaemia in a hyperosmolar state should not be aggressively corrected to avoid the potential for cerebral oedema with rapid reduction [9]. Finally, the combination of elevated sodium and glucose can lead to underestimating the actual water deficit. Our patient gained 18 kg over 9 days by the time eunatraemia was achieved, in contrast to the initial calculation of an 11 L water deficit.

## 4. Conclusion

We present a rare case of altered conscious state due to acute extreme hypernatraemia caused by hyperglycaemia and dehydration, with an initial corrected sodium level of 202 mmol/L. The patient was managed with the recommended correction rate of serum sodium concentration of 0.5 mmol/L/. He had almost complete neurological recovery with mild cognitive impairment by the time of hospital discharge. This supports the safety of a sodium correction of 0.5 mmol/L/hour in extreme hypernatraemia with concomitant hyperglycaemia.

## Figures and Tables

**Table 1:** Blood laboratory values over the initial 96 hours.

		Hours post presentation				
	7 days prior	0	24	48	72	96
Sodium (mmol/L)	141	191	182	171	162	152
Corrected Sodium (mmol/L)	141	202	190	175	163	153
Potassium (mmol/L)	4.9	3.8	4.4	4.9	4.2	3.9
Bicarbonate (mmol/L)	29	22	18	16	20	23
Chloride (mmol/L)	102	143	158	154	143	128
Urea (mmol/L)	5.4	24.7	23.0	21.7	17.9	16.0
Creatinine (*μ*mol/L)	107	200	164	185	139	112
Glucose (mmol/L)	6	31	22	18	7	9
pH	7.4	7.31	7.41	7.28	7.41	7.51
